# Diversity and evolution of leukotoxin operons in *Staphylococcus aureus*

**DOI:** 10.1128/msystems.01735-25

**Published:** 2026-04-20

**Authors:** Stephanie S. R. Souza, Marven J. Berlus, Cheryl P. Andam

**Affiliations:** 1 Department of Biological Sciences, University at Albany, State University of New York, Albany, New York, USA; University of Birmingham 1724, Birmingham, United Kingdom

**Keywords:** *Staphylococcus aureus*, leukotoxin, virulence, genome, evolution

## Abstract

*Staphylococcus aureus* is a globally significant and opportunistic pathogen equipped with a diverse arsenal of virulence factors, including bi-component leukotoxins that play a central role in immune evasion and tissue damage. Despite extensive functional characterization of individual leukotoxins, their diversity, phylogenetic distribution, and evolution remain underexplored. Here, we analyzed 1,779 complete *S. aureus* genomes to examine six leukotoxin operons: *hlgABC*, *lukED*, *lukAB*, *lukSF-PV*, *lukMF*', and *lukPQ*. Individual genomes carry anywhere between 1 and 4 operons, with 1,123 genomes (63.1%) carrying three operons. Only *hlgABC* and *lukAB* were detected at very high frequency (≥99%) and are thus considered core leukotoxins. Based on the different combinations of the six operons, 14 distinct leukotoxin profiles can be distinguished. The 11 sequence types that were most common (ST1, ST5, ST8, ST30, ST45, ST59, ST72, ST105, ST239, ST398, and ST1292) tend to carry one or two unique leukotoxin combinations. Genomes simultaneously carrying *hlgABC + lukED + lukAB* were common (*n* = 1,064 genomes). We also identified a genome carrying four copies of *lukAB*, with evidence of pseudogenization in the three plasmid-borne copies of *lukAB*. Of the 11 individual leukotoxin genes, *lukA*, *lukB*, *lukD*, *hlgB*, and *hlgC* experienced intragenic homologous recombination (all with *P* < 0.05). Only *lukS*, *lukF*, *lukE*, and *hlgA* exhibited Tajima’s *D* < −2.0, indicating a strong signal of purifying selection. Altogether, our results show that variable combinations of leukotoxin operons are differentially distributed across the species, with individual genes experiencing different levels of recombination and selection.

**IMPORTANCE:**

Leukotoxins are a key virulence factor of *Staphylococcus aureus*, helping the bacteria evade the host’s immune system. These toxins act as lytic molecules that directly target and kill a variety of immune cells. In our study, we show that six leukotoxin operons in *S. aureus* are combined in different ways within each genome, which may contribute to the pathogen’s ability to cause a myriad of diseases and colonize different tissue sites. Understanding the diversity and evolution of different leukotoxin genes will be critical to the development of anti-leukotoxin antibodies and immunization countermeasures that will effectively impede *S. aureus* infections.

## INTRODUCTION

*Staphylococcus aureus* is an opportunistic pathogen that causes a wide range of infections, ranging from superficial skin lesions to life-threatening systemic infections (1). A major public health threat, *S. aureus* is the top bacterial cause of death in 135 nations and is the only pathogen associated with >1 million deaths and 34 million years of life lost globally, according to the 2019 Global Burden of Disease Study (2). A significant challenge in combating *S. aureus* infections is the emergence and spread of antimicrobial-resistant strains, most notably multidrug-resistant and methicillin-resistant *S. aureus* (MRSA), which limit treatment options and exacerbate clinical outcomes (3). MRSA infection is associated with significantly higher mortality, prolonged hospital stays, and increased medical expenses (4, 5). From 1990 to 2021, the global burden of disease attributed to MRSA doubled from 57,200 to 130,000 and will continue to accelerate if diverse public health interventions are not urgently implemented (6).

*S. aureus* is notable for its large arsenal of virulence factors, including an array of toxins and enzymes (7). An important group of staphylococcal virulence factors is the bi-component, pore-forming leukotoxins that play a crucial role in immune evasion and tissue damage (8, 9). Leukotoxins consist of two distinct protein subunits—designated as S (slow-migrating) and F (fast-migrating) components—that oligomerize to form β-barrel pores in the membranes of host immune cells (10, 11). The S component typically mediates receptor binding, while the F component facilitates pore formation (10, 11). Leukotoxins primarily target leukocytes, which include neutrophils, monocytes, macrophages, and dendritic cells, thereby disrupting both innate and adaptive immune responses (8, 9). Formation of pores within cellular lipid bilayers ultimately leads to the leakage of divalent cations (8, 9). The release of ions impairs critical cellular homeostasis, consequently causing cell lysis (11, 12).

The most well-known leukotoxins secreted by human-associated *S. aureus* are Panton-Valentine leukocidin (PVL; LukSF-PV), gamma-hemolysin AB (HlgAB) and CB (HlgCB), leukocidin ED (LukED), and leukocidin AB (LukAB, also known as LukGH) (8, 9). Two other leukotoxins—leukocidin MFʹ (LukMFʹ) and leukocidin PQ (LukPQ)—are typically related to *S. aureus* strains associated with bovine and equine infections, respectively (13, 14). Except for the independently transcribed *hlgA*, the S and F subunits of a given toxin pair are co-transcribed from a single promoter (8). Interactions of leukotoxins with host cell receptors (C5aR1, CCR5, CXCR1, and CD11b) are highly specific, which contribute to their cell-type tropism (8, 9). While leukotoxins are named after leukocytes (white blood cells) they target, gamma-hemolysin and LukED are known to also lyse red blood cells, targeting the same erythroid receptor DARC (Duffy antigen receptor for chemokines) (15, 16). The expression of leukotoxin genes is tightly regulated through the Agr quorum-sensing and SaeRS host-responsive systems, with their production induced during contact with neutrophils or blood, thus ensuring that these toxins are deployed precisely during infection (17, 18). Leukotoxins have been proposed as promising targets for therapeutic intervention and vaccine development (19, 20). Innate immune evasion genes, including leukotoxins, in *S. aureus* have been reported to be lineage specific (21), yet a comprehensive study of their diversity and distribution across the species is lacking.

Here, we analyzed 1,779 complete *S. aureus* genomes to describe the phylogenetic distribution of five leukotoxin operons (*hlgABC*, *lukED*, *lukAB*, *lukSF-PV*, *lukMF'*, and *lukPQ*). Altogether, our results show that variable combinations of leukotoxin operons are differentially present across the species, with individual genes experiencing different levels of recombination and selection. Characterizing the diversity of leukotoxin operons is imperative in understanding the genetic basis of the virulence of *S. aureus*.

## RESULTS

### Leukotoxin operons are differentially distributed among *S. aureus* genomes

**Fig 1:**
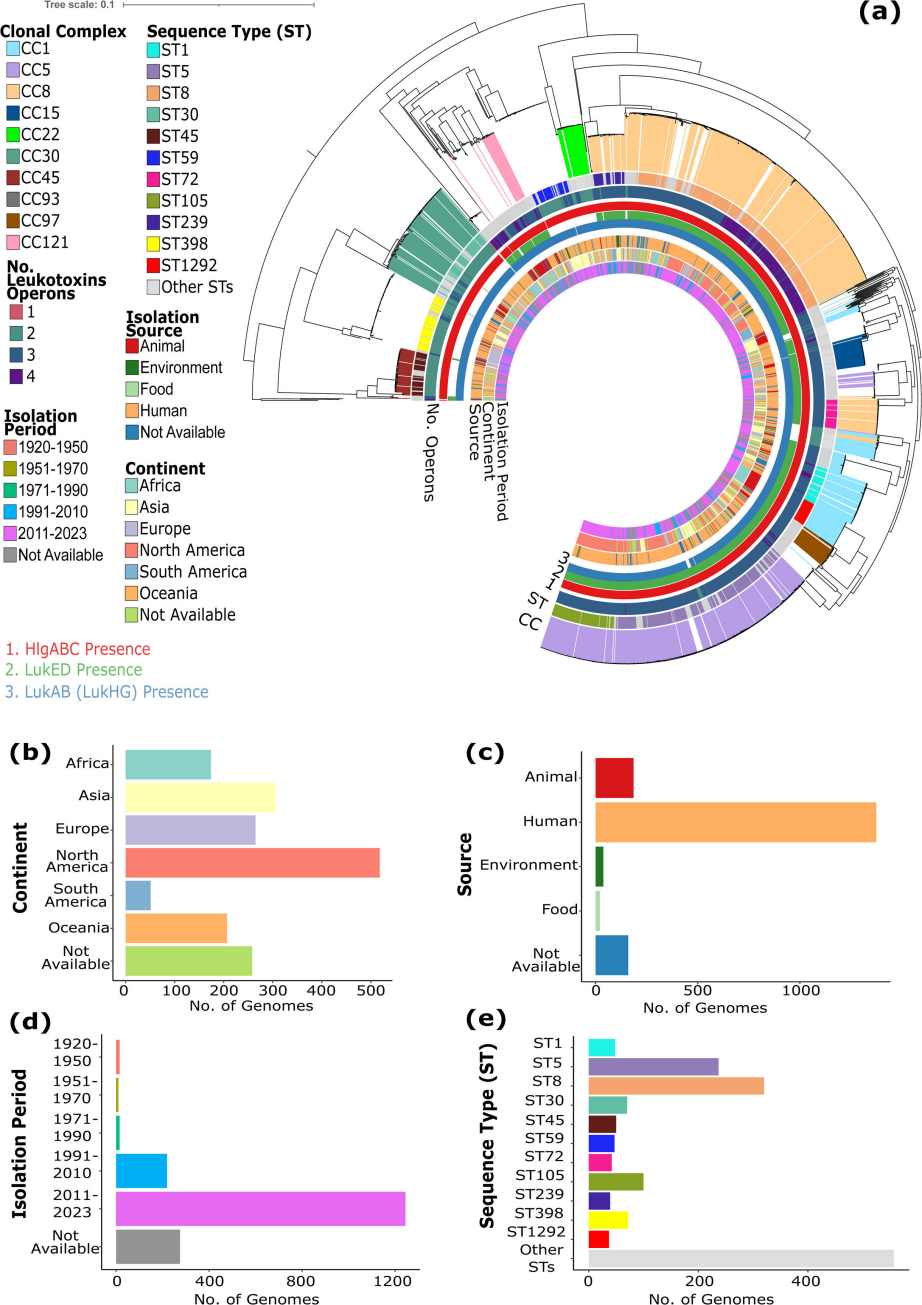
Population structure of 1,779 *S. aureus* genomes. (**a**) Maximum likelihood tree showing the phylogenetic relationships of the 1,779 *S. aureus* genomes. Tree scale represents the number of nucleotide substitutions per site. The tree is rooted at its midpoint. Colored lines extending out of the branches of the tree represent the clonal complex (CC). The outer rings (from outer to inner) represent the sequence types (STs), number of operons per genome, presence of the three most common leukotoxin operons (*hlgABC*, *lukED*, and *lukAB*), source, continent, and isolation period. For visual clarity, only the 11 most frequent STs are shown, and years are grouped into decades. Distribution of genomes by (**b**) geographic origin, (**c**) source of isolation, (**d**) temporal period of isolation, and (**e**) STs. For panels **b–e**, the colors of bars are identical to those in panel a. Detailed information on the genomic features and associated metadata of individual genomes is presented in Table S1.

Our data set consisted of 1,779 complete *S. aureus* genomes that are phylogenetically, geographically, and ecologically represented (Fig. 1a). Geographic metadata was available for 85.5% of our data set (1,521 genomes). Genomes from North America were overrepresented (518 genomes, 29.1%), followed by Asia (295 genomes, 16.6%), with South America contributing the fewest genomes (51 genomes, 2.9%; Fig. 1b). The majority of genomes were derived from human sources (1,374 genomes, 77.2%), with additional genomes originating from animals (186 genomes, 10.4%), environmental samples (38 genomes, 2.1%), and food sources (21 genomes, 11.8%; Fig. 1c). The temporal distribution of the data set spanned over a century (years 1924–2023), with most genomes (1,245 genomes, 70%) collected between 2011 and 2013 (Fig. 1d).

We identified 206 distinct sequence types (ST) and 10 clonal complexes (CC). A total of 123 STs were represented by only one genome. The most prevalent STs—ST1, ST5, ST8, ST30, ST45, ST59, ST72, ST105, ST239, ST398, and ST1292—collectively accounted for 59.7% of the data set (1,062 genomes; Fig. 1e). The most common MRSA lineages (22, 23) in our data set were ST5 (CC5), ST8 and ST239 (both from CC8), ST22 (CC22), ST36 (CC30), and ST545 (CC45). Genomes belonging to low-frequency STs were grouped as “Other STs,” and these represented 31.3% of the data set (557 genomes). A total of 160 genomes could not be typed using the methods applied. Among the dominant STs, ST8 was the most common (320 genomes), followed by ST5 (237 genomes) and ST105 (100 genomes).

**Fig 2:**
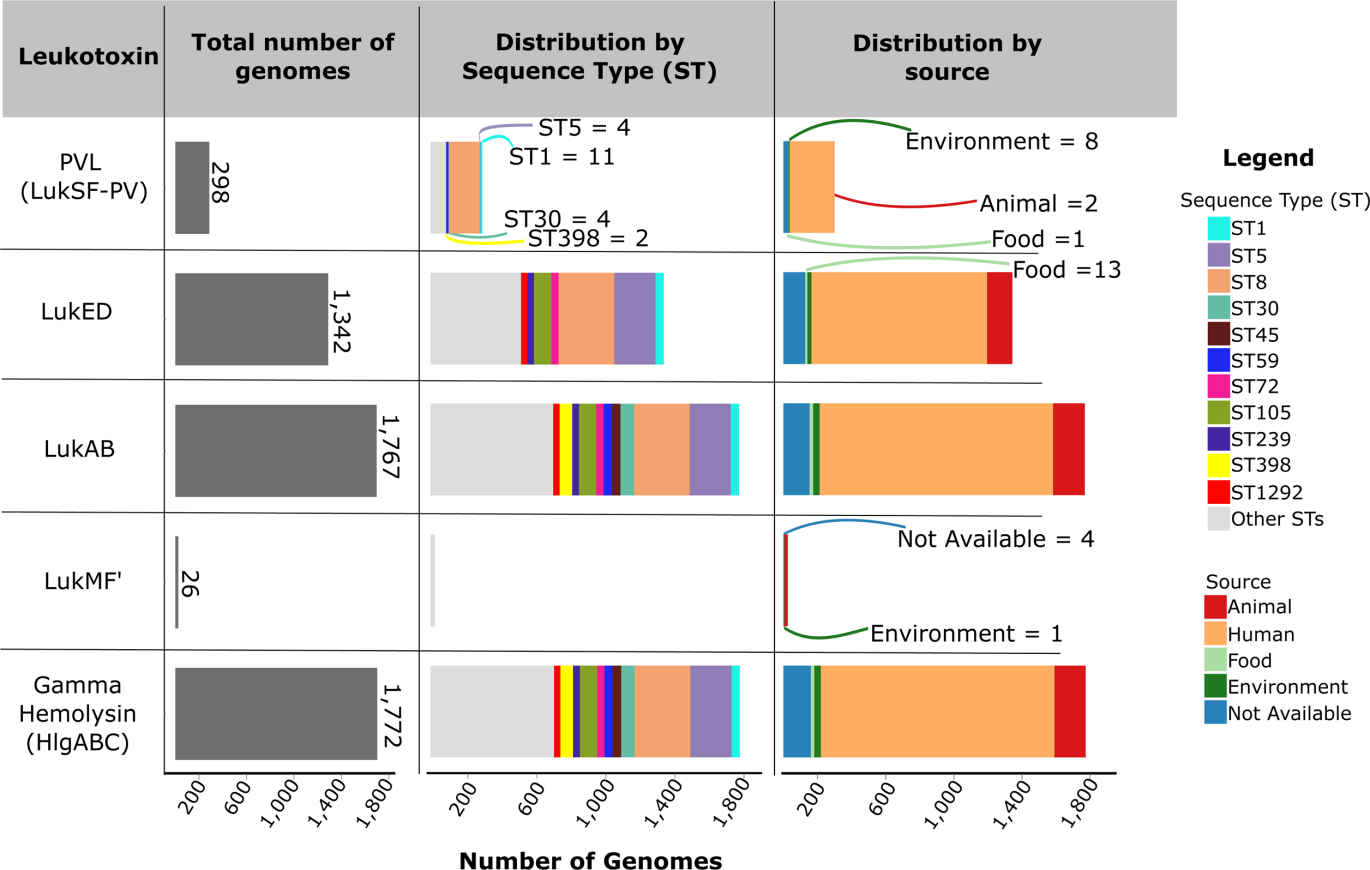
Distribution of the five leukotoxin operons. Bar plots summarize the presence of five leukotoxin operons across the data set, showing their total counts, distribution by sequence types (STs), and isolation sources. For visual clarity, only the 11 most frequent STs are shown. Colors of bars are identical to those in Fig. 1a. *lukPQ* is not shown because it was detected in only three genomes (one from ST1 [CC1] and two from low-frequency STs). Of these three *lukPQ*-carrying genomes, two originated from animal sources, whereas one did not have information on its ecological source.

Of the six leukotoxin operons, only *hlgABC* and *lukAB* were detected at high frequency (≥99%) in the 11 dominant STs (Fig. 2 and Fig. S1). *hlgABC* was present in 1,772 genomes (99.6% of data set). This operon encodes two functional bicomponent toxins—HlgAB and HlgCB—formed by pairing the shared *hlgB* gene with either *hlgA* or *hlgC*, with each toxin employing specific chemokine receptors (24). Although *hlgA* is transcribed separately, the three genes are commonly found together in the genome (8), consistent with previous observations in other *S. aureus* populations (25–27). Seven genomes carried only *hlgAB* or *hlgCB*, but not all three genes. *hlgAB* was detected in two ST152 genomes and one untyped genome. *hlgCB* was present in four genomes belonging to ST4581, ST3576, ST7619, and ST59, and which originated from animal sources (three genomes) and one from a human source (Table S1). On the other hand, the *lukAB* operon was present in 1,767 genomes (99.3%) across all STs and ecological sources. Genomes missing one of the two l*ukAB* genes, considered in this case as incomplete, were rare; one genome was missing *lukA*, while another genome was missing *lukB* (Table S1). These genomes were both from human sources and belonged to ST152 and ST582, respectively. There were 10 genomes in which this operon was completely absent, of which seven genomes were members of ST228 (CC5).

The *lukED* operon was also relatively common. It was detected in 1,342 genomes (75.4%; Fig. 2 and Table S1). Although it was present in 159 different STs, *lukED* was completely absent in genomes from ST30 (CC30), ST45 (CC45), ST59, and ST398. We also identified three genomes that were missing a *lukE* gene but not the *lukD* (Table S1). These genomes were exclusively isolated from human sources and belonged to ST26, ST152, and an untyped genome.

In contrast with the wide distribution of *hlgABC*, *lukAB*, and *lukED*, we identified *lukSF-PV* (PVL) in 298 genomes, of which 59% (176 genomes) belonged to ST8 (Fig. 2 and Table S1). It was also found in 13 genomes from ST59, 11 genomes from ST1, 4 from ST5 and ST30, and 2 from ST398 as well as in 24 low-frequency STs. *lukSF-PV* was most common in human-derived genomes (260 genomes). The operon *lukMF'* was present in 26 genomes, all belonging to low-frequency STs. The most common *lukMF'*-carrying genomes belong to ST133 (eight genomes). None of the human-derived genomes carried *lukMF'*, whereas 21 of the 26 genomes originated from animal sources. We identified three genomes with *lukF'* but lacking *lukM*. These three genomes are members of ST1, ST8750, and ST350. Finally, the least common leukotoxin operon is *lukPQ*, which was detected in three genomes (one from ST1 [CC1] and two from low-frequency STs). Of these three genomes, two originated from animal sources, whereas one did not have information on its ecological source.

The leukotoxin genes reported above were identified using thresholds of at least 80% sequence identity and 80% sequence coverage against our database of reference genes. However, even when using more stringent cutoffs in sequence similarity (90% sequence identity and 90% sequence coverage; 95% sequence identity and 95% sequence coverage), we found minimal or no change in the numbers of genomes carrying the individual leukotoxin genes (Table S2).

### Different genomes carry assorted combinations of leukotoxin operons

**Fig 3:**
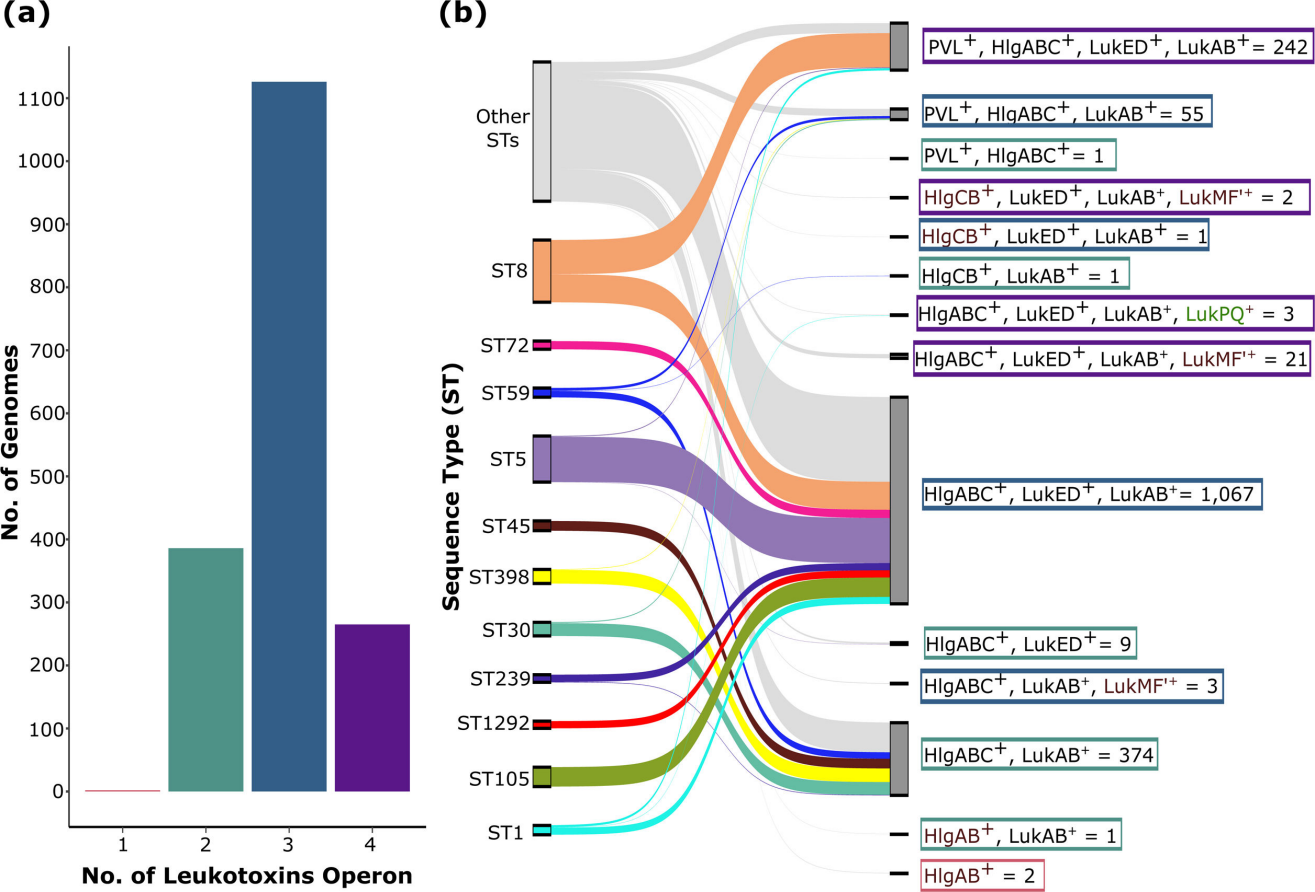
Diversity of leukotoxin profiles. (**a**) Distribution of genomes according to the number of leukotoxin operons found per genome. (**b**) Sankey plot showing the distribution of the 14 leukotoxin profiles among the STs. Leukotoxin profiles are based on the different combinations of the six operons. + means the operon is present. The number in boxes indicates the number of genomes. Colored boxes of the leukotoxin profiles indicate the number of operons present (4, purple; 3, blue; 2, green; 1, red).

All genomes analyzed in this study harbored at least one leukotoxin operon (range: 1–4 operons per genome). Yet, not a single genome contained all six leukotoxin operons. Most genomes carry three operons (1,126 genomes; Fig. 3a and Table S1). The genome with only one operon belonged to ST59 derived from a human source. Based on the different combinations of the five operons, we classified the genomes into 13 distinct leukotoxin profiles (Fig. 3b). Of these, three profiles included the concomitant presence of four operons, while three profiles encompassed three operons. Profiles consisting of two distinct operons were highly variable and comprised five different profiles. The most common leukotoxin profile consisted of genomes simultaneously carrying *hlgABC + lukED + lukAB*, which was found in 1,067 genomes (Fig. 3b). This profile was distributed across 139 different STs and was observed in all genomes from ST72, ST1292, and ST105. Yet, none of the ST59 genomes harbored this profile. The second most common profile included genomes harboring *hlgABC + lukAB* (*n* = 374 genomes), which were found in 45 different STs and were the only profile present in all genomes from ST398 and ST45. Yet none of the ST1 genomes harbored this profile. For some STs, their member genomes tend to carry specific combinations of leukotoxin operons more frequently. For example, ST72, ST1292, and ST105 consistently carried the profile combining *hlgABC + lukED + lukAB*, while ST398 and ST45 only had the combination of *hlgABC + lukAB. lukED* was notably absent in ST30, ST45, ST59, and ST398. Eight profiles were found in only 1–3 genomes, mostly from low-frequency STs (collectively referred to as “Other STs” in Fig. 2b). These less common profiles include the ones associated with *lukMF'* and *lukPQ*.

ST5 and ST8 are the dominant STs worldwide (22, 23). Examining their leukotoxin profiles, we found that most ST5 genomes carried the profile containing *hlgABC + lukED + lukAB* (232 out of 237 genomes; Table S1). On one hand, ST8 genomes carried only two profiles differentiated by the presence or absence of the *lukSF-PV* in combination with *hlgABC + lukED + lukAB* (Fig. 3b). Among the 320 ST8 genomes, 176 genomes carried the profile containing *lukSF-PV*, while 144 genomes lacked *lukSF-PV*. Further analysis revealed that the presence or absence of PVL was associated with genomes carrying SCC*mec* (Fig. S2 and Table S3). Of the 176 ST8 genomes carrying *lukSF-PV*, 167 genomes also co-harbored SCC*mec* (94.9%), of which 155 genomes carried cassette type IVa (92.8%). In contrast, among the 144 ST8 genomes lacking *lukSF-PV*, 51.4% (74 genomes) also lacked the SCC*mec* element.

### Multiple copies of *lukAB* are present in the chromosome and plasmid of a single genome

**Fig 4:**
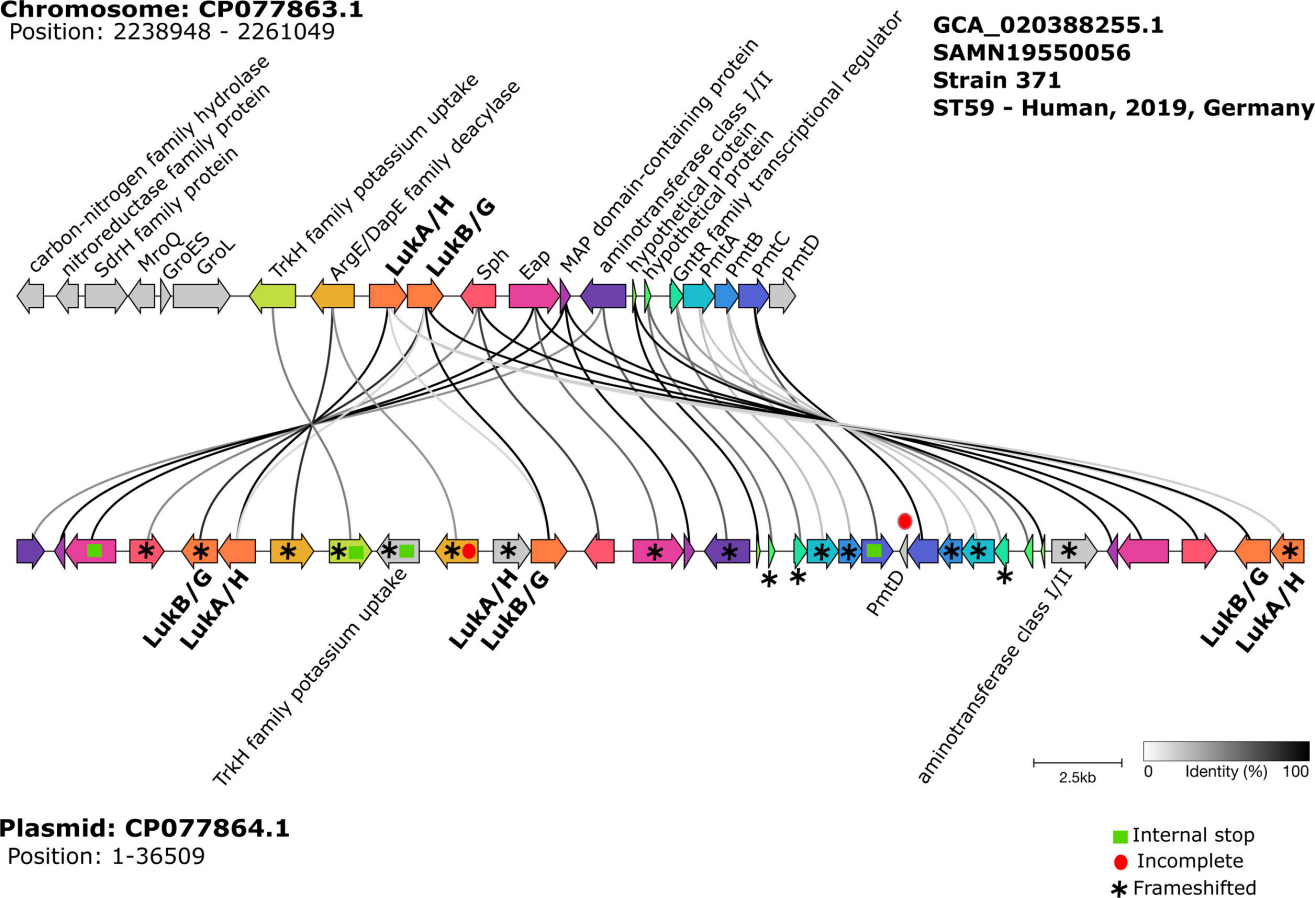
Genomic neighborhood of the four copies of *lukAB* operon in *S. aureus* strain 371 (accession number: GCA_020388255.1). Top: The chromosomal region includes 10,000 bp flanking sequences upstream and downstream of *lukAB*. Bottom: Three copies of *lukAB* in the plasmid. Grayscale lines connecting genes between the chromosome and the plasmid indicate sequence identity (%). *lukAB* is also known as *lukHG*. Colored arrows represent genes that are present in both the chromosome and the plasmid.

Nearly all genomes in our data set carried leukotoxins on the chromosome. We only detected two instances of *lukAB* located on plasmids, both in *S. aureus* genomes derived from human sources in Germany in 2019 (Table S1). One genome with plasmid-associated *lukAB* belonged to ST8 (accession number: GCA_020388395.1), while the second genome belonged to ST59 (accession number: GCA_020388255.1). This second genome was intriguing because it harbored four copies of the *lukAB* operon (Fig. 4). One *lukAB* copy is on the chromosome (*lukA:* 2,248,948–2,250,003 bp; *lukB:* 2,250,025–2,251,041 bp), while the remaining three were located on a single plasmid (*lukA* plasmid copy 1: 5,706–6,761 bp, copy 2: 13,514–14,568 bp, and copy 3: 35,578–36,509 bp; *lukB* plasmid copy 1: 4,668–5,684 bp, copy 2: 14,590–15,606 bp, and copy 3: 34,540–35,556 bp). Further plasmid typing analysis identified this plasmid as a non-mobilizable plasmid.

To further examine this unusual genome, we extracted 10,000 bp regions flanking both sides of the chromosomal *lukAB* and compared it against the genetic neighborhood of the three *lukAB* copies on the plasmid (Fig. 4). Sequence similarity among the chromosomal copy and plasmid copies ranged from 30% to 100%, although all have the reference *lukAB* genes as their top hits. Synteny analysis revealed high genetic similarity among the neighboring genes of the *lukAB* operons in the chromosome and plasmid. The three plasmid copies of *lukAB* included a repeated segment containing the same flanking genes as the chromosomal copy. Genes in the flanking regions of the chromosomal and plasmid copies of *lukAB* consistently included *trkH*, *pmtA-D*, *argE/dapE* family, *sph* (*hlb* in *S. aureus*), and *eap*. These genes are known to be involved in various cellular functions: *trkH* is part of the Ktr potassium uptake systems (28); *pmtA-D* are involved in toxin export (29); *sph* encodes a sphingomyelinase that breaks down the lipid component of the cell membrane (30); and *eap* is an extracellular adherence protein (31). However, within the plasmid region of *lukAB* were several genes, including *lukAB*, that showed evidence of having experienced a frameshift mutation, incomplete sequence, or containing internal stop codons. These features suggest that the chromosomal copy of *lukAB* may have been integrated multiple times into the plasmid and is likely undergoing pseudogenization or gene decay, likely due to redundant functionality.

### Leukotoxin genes differentially experienced recombination and selection

To determine if the leukotoxin genes have experienced recombination, we performed a Pairwise Homoplasy Index (PHI) test (32) for each gene (Table 1). We found variable results from the recombination test among the leukotoxin genes. Of the 11 individual leukotoxin genes, we found significant evidence of recombination in *lukA*, *lukB*, *lukD*, *hlgB*, and *hlgC* (all with *P* < 0.01). The genes that make up *lukSF-PV* and *lukMF'* did not show significant evidence for recombination.

**TABLE 1:** Results of the Pairwise Homoplasy Index (PHI) test and neutrality analysis for each leukotoxin gene.^*a*^

	PHI test		Neutrality test	
Gene	Recombination detected	*P* value	Nucleotide diversity (π)	Tajima’s *D*
*lukS*	No	1	0.001637	−2.48
*lukF*	No	1	0.001197	−2.32
*lukE*	No	0.06451	0.12912	−2.04
*lukD*	Yes	0.0106	0.012991	−1.95
*lukA/H*	Yes	<0.0001	0.042947	0.6
*lukB/G*	Yes	<0.0001	0.048495	0.79
*lukM*	No	1.0	0.001902	−1.29
*lukF'*	No	1.0	0.031405	−1.16
*hlgA*	No	0.2867	0.005981	−2.02
*hlgB*	Yes	5.95E-06	0.02101	−0.7
*hlgC*	Yes	5.86E-10	0.02582	−1.13

^*a*^ Alternating gray and white rows indicate genes that make up an operon. *lukPQ* was not included in this analysis due to the low number of genomes (*n* = 3) that contain this operon.

We next sought to determine whether the pattern of mutations found in a gene in the population follows the assumptions of neutrality, i.e., if there has been selection for or against, using Tajima’s *D* statistic (33). A value of 0 means the gene being tested is evolving neutrally per the mutation-drift equilibrium (33). The range of Tajima’s *D* values was −2.48 to 0.79 (Table 1). Only *lukA* and *lukB* had positive values, indicating an excess high-frequency variation, thus suggesting potential balancing selection. The rest of the leukotoxin genes had values less than 0, indicating an excess of low-frequency polymorphisms consistent with purifying selection. Values that are lower than −2 and greater than 2 are typically considered strong evidence of selection, i.e., a gene is not evolving neutrally (34, 35). Thus, in this case, only *lukS*, *lukF*, *lukE*, and *hlgA*, all with *D* < −2.0, exhibit strong evidence of purifying selection.

## DISCUSSION

Bi-component leukotoxins enable *S. aureus* to resist the actions of the host immune system and are therefore major players in its pathophysiology. Here, we analyzed 1,779 complete *S. aureus* genomes to describe the phylogenetic distribution of six leukotoxin operons (*hlgABC*, *lukED*, *lukAB*, *lukSF-PV*, *lukMF'* and *lukPQ*). Our main findings are that (i) leukotoxin operons can be categorized as core (*hlgABC* and *lukAB*) and accessory elements (*lukED*, *lukSF-PV*, *lukMF', lukPQ*), (ii) various combinations of leukotoxin operons are distributed among strains, and (iii) individual leukotoxin genes experience different levels of recombination and selection. Characterizing the diversity and phylogenetic distribution of leukotoxin operons is imperative in understanding the range of *S. aureus* infections and the underlying genetic causes. Our findings will be useful in the development of anti-leukotoxin antibodies and immunization countermeasures that will help impede *S. aureus* infections.

The nearly universal presence of *hlgABC* and *lukAB* (>99% of genomes) suggests that any one strain carries with it the potential to cause disease, thus reflecting the opportunistic nature of *S. aureus. hlgABC* encodes two distinct toxins (HlgAB and HlgBC) that differentially attack phagocytes by employing specific chemokine receptors (24). Although carrying either *hlgAB* or *hlgBC* only is not uncommon, having all three *hlg* genes in the same genome is beneficial to the cell as it can result in the production of a mix of HlgAB and HlgBC pores and thus substantially increase the number of active toxins present (36). LukAB is known to be ubiquitous in clinical *S. aureus* (37), likely due to its multi-functional nature. It targets human neutrophils (38), facilitates bacterial survival inside phagocytes (39), and hinders the development of adaptive immunity by targeting dendritic cells (40). In our study, these two operons were found in the 11 dominant STs, some of which are known to be members of globally important epidemic and virulent MRSA clones (22, 23) CC1, CC5, CC8, CC30, CC45, CC59 (causes community-associated infections [41]), CC239 (predominant in Asia [42]), and CC398 (livestock-associated [43]). The presence of these two operons in rare STs is a cause for concern. This is because the history of antimicrobial resistant *S. aureus* is characterized by the occurrence of epidemic waves initiated by one or a few successful clones, which replace previously dominant clones (44). Notably, the first pandemic wave occurred in the 1940s and was caused by a penicillin-resistant lineage named phage-type 80/81 (Φ80/81) that produced PVL (45, 46). It is therefore not inconceivable that any one of these less common CCs, already carrying *hlgABC* and *lukAB*, may become part of future waves of antimicrobial resistance. Also intriguing was the case of the one genome carrying three plasmid-borne mutated copies of *lukAB* that we have observed. Pseudogenization or decay of these extra *lukAB* copies is likely due to redundant functionality. Similarly, gene decay of the leukotoxin operon in the genus *Mannheimia* has been previously reported (47).

The genes encoding the accessory leukotoxins are known to be in close proximity to mobile genetic elements. *lukSF-PV* is located on temperate phages mainly ϕSa2 (ϕSLT) (48, 49), while *lukMF′* is on the phage ϕSa1 (ϕPV83) (50). *lukED* is within the *S. aureus* pathogenicity island vSaβ (51) that relies on helper phages to be mobilized (52, 53). These mobile genetic elements mediate horizontal gene transfer and gene loss, which may explain the three leukotoxins’ disparate phylogenetic distribution and abundance. What was certainly intriguing is our observation that the five operons are found in different combinations (profiles) among genomes, with certain STs primarily dominated by one or two leukotoxin combinations. These combinations likely reflect the variation in cytotoxic potential, virulence features, and types of diseases associated with distinct STs. A noteworthy consequence of this mix-and-match scheme is that distinct leukotoxins can work together, as in the case of PVL and gamma-hemolysin AB in biofilms in chronic infections that together induce extracellular trap formation and evade neutrophil-mediated killing (54). Yet, a previous study has shown that PVL and LukED tend to antagonize each other, with PVL inhibiting LukED-mediated lysis of erythrocytes by forming complexes with LukED, thus impairing pore formation (55). In our study, we found the combination *lukSF-PV + hlgABC + lukED + lukAB* in 242 genomes, although no genome carried only *lukSF-PV + lukED*. Antagonism between PVL and LukED is likely compensated by the presence of other types of leukotoxins produced by a cell. Moreover, leukotoxins are also known to have synergistic effects with other virulence factors such as alpha-toxin and phenol-soluble modulin to disarm immune defenses (56, 57). Another major advantage of the variable combinations of leukotoxins is that, except for the pre-assembled LukAB, a leukotoxin can form a non-cognate pairing of the S-component with an F-component of another leukotoxin, for example, LukE/HlgB and HlgA/LukD (9). Thus, any one genome can substantially increase its pool of unique leukotoxins, consequently broadening its cytotoxic potential and ability to target a medley of cellular receptors. This versatility allows *S. aureus* to attack a wide range of tissues and host immune cells, including neutrophils, monocytes, macrophages, and dendritic cells, thereby causing the disruption of innate and adaptive immune responses (9).

Leukotoxin genes are subject to recombination and selection, although they were highly variable among individual genes. Recombination within genes could lead to sequence diversity that may cause slight modifications affecting the toxin assembly, stability, or the efficiency of pore formation in host cell membranes. Similarly, novel superantigens produced by *S. aureus*—enterotoxin-like toxins U2 and V—arose via recombination within the enterotoxin gene cluster, likely facilitated by associated insertion sequences and transposases (58). The authors demonstrated that these two toxins exhibited superantigen activity, stimulating polyclonal T-cell proliferation (58). Recombination of toxin-encoding genes has been reported in other bacterial pathogens. For example, four variants of the MARTX(Vv) cytotoxin produced by the foodborne pathogen *Vibrio vulnificus* arose through recombination in the *rtxA1* gene (59). The *Vibrio* toxin variants have different arrangements of effector domains and exhibit variable levels of potency in humans versus oysters, which are their natural host in aquatic environments (59). Intragenic recombination in the toxin B (TcdB) produced by *Clostridioides difficile* generates sequence variants that contribute to hypervirulence and severe pathology (60, 61) as well as toxin-receptor recognition (62). Co-evolution between host and pathogen drives high virulence and selection favoring elevated copy number of the nematocidal toxin genes encoded by *cry14Aa1* and *cry21Aa2*, as demonstrated in the nematode host *Caenorhabditis elegans* and its natural pathogen *Bacillus thuringiensis* (63). Our results mirror those reported in the leukotoxin genes *lktC*, *lktB*, and *lktD* of the bovine pathogen *Mannheimia haemolytica*, whereby different *lkt* genes vary in terms of the frequency of intragenic homologous recombination and selection (64). Loci with high sequence similarity, as in the case of the leukotoxin genes, can carry out non-reciprocal intrachromosomal homologous recombination (similar to gene conversion in eukaryotes) whereby short tracts of sequence are copied from one homolog to another (65). In *M. haemolytica*, it has been postulated that host switching of the bovine strains from cattle to sheep may have been responsible for driving these evolutionary processes in its leukotoxin operon (64). We can only speculate if a similar scenario exists in *S. aureus*, which is also a multi-host pathogen (66), and future work is needed to test this. Nonetheless, our leukotoxin study contributes to current knowledge on the evolutionary processes that shape toxin diversity in bacterial pathogens.

A future line of investigation is examining the cytotoxic effects of the 13 different leukotoxin combinations (which we refer to as profiles) in different genetic backgrounds of *S. aureus* and in different host species, which will bring profound insights into the multi-host adaptive quality of *S. aureus*. Moreover, it is worth exploring the diversity and functions of leukotoxin-encoding genes in other *Staphylococcus* species, especially with respect to host adaptation. The leukotoxin LukSF-I in *Staphylococcus pseudintermedius* is encoded on a degenerate prophage and has the capacity to target both canine and human leukocytes (67, 68). Intriguingly, *S. pseudintermedius* LukS-I is closely related to *S. aureus* LukE and LukP, while LukF-I is closely related to *S. aureus* HlgB (67). Bi-component leukotoxin-like toxins have also been found in other bacterial species (69). Last, reconstructing the origins and ancient evolutionary history of staphylococcal leukotoxins and those from other species will be essential to elucidate the processes that shape their diversity, how cognate and non-cognate pairings had evolved, and the mutational intermediates that descended from the ancestral leukotoxin.

We acknowledge the limitations of our study. First, the data set was limited to complete genomes available in the National Center for Biotechnology Information (NCBI), which certainly leads to sampling bias because strains from clinical human sources tend to be overrepresented. Second, although we assessed the presence of leukotoxin operons using sequence identity and coverage thresholds, we did not evaluate the functional expression and regulation of these genes. The presence of a gene does not guarantee its transcriptional activity or its role in virulence *in vivo*. Future studies that include transcriptomic or proteomic data will be necessary to confirm their functional relevance. This will also be useful in understanding the importance of having more than one leukotoxin operon in a genome, especially related to cellular targets, host immune response, specificity in infection sites, and interactions among leukotoxins. Nonetheless, this study provides an initial comprehensive overview of the diversity, distribution, and evolution of leukotoxin operons in *S. aureus*. Our findings bring important insights that will help advance and support the development of novel anti-staphylococcal vaccines and therapeutic agents targeting leukotoxins.

## MATERIALS AND METHODS

### Genome collection

We retrieved a total of 1,806 complete genome sequences of *S. aureus* from the NCBI RefSeq Database in January 2024. Genome quality was assessed using the programs QUAST (70) and CheckM (71). All genomes were compared against each other using the program fastANI v.1.32, implementing a ≥95% average nucleotide identity (ANI) threshold to ensure that the genomes are all of the same species (72). Genomes with <90% completeness and >5% contamination and/or ANI values <95% were excluded. After filtering based on these criteria, we obtained a total of 1,779 genomes for all downstream analyses (Table S1). Our use of complete genomes and strict filtering criteria is critical to reduce the impact of missing genes in incomplete, fragmented, and poorly sequenced genomes.

### Phylogenetic tree reconstruction

Complete genomes were annotated using Prokka v.1.14.6 (73). We characterized the entire set of gene families (collectively called pan-genome [74]) using Panaroo v.1.3.3 (75). We defined core genes as those present in ≥95% of the genomes (i.e., at least 1,690 genomes), whereas the accessory genes were those present in <95% of the data set. Sequence alignment of individual genes was carried out using PRANK (76). Using the concatenated alignment of the 2,115 core genes, we extracted single-nucleotide polymorphisms (SNPs) using SNP-sites v.2.5.1 (77). The aligned core SNPs were used to build a maximum likelihood phylogenetic tree using FastTree v.2.1 (78) with a generalized time reversible model (79) of nucleotide substitution, a Gamma distribution of rate heterogeneity, and 100 bootstrap replicates. The phylogenetic tree was visualized using the online platform Interactive Tree of Life (80). We used the same phylogenetic reconstruction method described above to build a phylogenetic tree of ST8 genomes.

### *In silico* sequence typing and detection of genetic elements

The ST of each genome was determined using the program mlst v.2.19.0 (https://github.com/tseemann/mlst), which uses unique allele combinations of seven single-copy housekeeping genes (*arcC*, *aroE*, *glpF*, *gmk*, *pta*, *tpi*, and *yqiL*) (81) in the *S. aureus* PubMLST database (82). CCs were determined based on PubMLST classification. We screened all genomes for the presence of operons that encode the following bi-component leukotoxins: PVL (*lukSF-PV*), γ-hemolysin (*hlgABC*), LukED (*lukED*), LukAB (*lukAB*, also known as *lukHG*), LukMF' (*lukMF'*)*,* LukPQ (*lukPQ*) (9). We first constructed a custom database in Abricate v.1.0.0 (https://github.com/tseemann/abricate) that contains reference sequences for the genes listed above. The accession numbers for the genes used to construct the custom database are listed in Table S4. We applied a minimum threshold value of ≥80% sequence identity and ≥80% sequence coverage for comparing query sequences with the sequences in the database. We also increased the stringency of our detection methods to include ≥90% sequence identity and ≥90% sequence coverage, as well as ≥95% sequence identity and ≥95% sequence coverage (Table S2). Only the top significant hits were considered. Only genomes carrying both components of an operon were considered positive for the presence of the leukotoxin. We also screened the genomes for the presence and type of SCC*mec* (staphylococcal chromosomal cassette *mec*) using the sccmec v.1.2.0 (https://github.com/rpetit3/sccmec).

### Recombination and selection analysis

To investigate the extent of within-gene homologous recombination, we estimated the PHI (32) separately for individual genes of each leukotoxin. We did not include *lukPQ* in this analysis due to the low number of genomes (*n* = 3) that contain this operon. The nucleotide sequences were first aligned using MUSCLE (83) implemented on the MEGA11 software (84) with default parameters for clustered methods and gap penalty. The PHI test calculates a pairwise incompatibility score of each site in a global sequence alignment, as well as the cumulative probability under a normal distribution obtained from the expected mean and variance of the PHI statistic. The significance of the PHI statistic is determined using a permutation test, where under the null hypothesis of no recombination, sites along the alignment are randomly permuted to generate the null distribution of PHI. *P* values < 0.05 indicate a significant presence of recombination. We ran the PHI test implemented on the SplitsTree program (85). To assess evidence of selection and nucleotide diversity within each leukotoxin gene alignment, we also estimated the neutrality statistic Tajima’s *D* statistic (33) using the MEGA11 software with default parameters. Tajima’s *D* measures the allele frequency distribution of nucleotide sequence data and tests the null hypothesis that sites evolve neutrally in a constant-size population (33).

### Synteny-based characterization of multiple *luk*AB copies

Based on initial *in silico* screening, we observed the presence of four copies of *lukAB* in a single ST59 genome (GCA_020388255.1), with one copy located in the chromosome and three copies on a single plasmid. To visualize the genomic neighborhood and assess the organization of these multiple copies of *lukAB*, we examined their gene synteny using Clinker v.0.0.31 (86). We extracted 10,000 bp regions from both upstream and downstream of the chromosomal copy of *lukAB*. We utilized the GenBank file of the entire plasmid containing *lukAB*. The plasmid was typed using MOB-typer v.3.1.0 module on MOB-suite (87) to determine its mobility and replicon type.

## Data Availability

The data set supporting the conclusions of this article is included within the article and its supplementary files. Genome sequence data of *S. aureus* are publicly available in the Short Read Archive database of NCBI. BioSample accession numbers for each genome are listed in Table S1.

## SUPPLEMENTAL MATERIAL

Supplemental figures msystems.01735-25-s0001.pdf Figures S1 and S2.

Supplemental tables msystems.01735-25-s0002.xlsx Tables S1-S4.

ASM does not own the copyrights to Supplemental Material that may be linked to, or accessed through, an article. The authors have granted ASM a non-exclusive, world-wide license to publish the Supplemental Material files. Please contact the corresponding author directly for reuse.
