# Supplementary material for: Diversity and evolution of leukotoxin operons in Staphylococcus aureus
Source: mSystems. 2026 Apr 20;11(5):e01735-25. doi: 10.1128/msystems.01735-25 (PMC13185589; doi:10.1128/msystems.01735-25)
Supplement: Supplemental figures — Figures S1 and S2. [file msystems.01735-25-s0001.pdf]

Supplementary information for

Diversity and evolution of leukotoxin operons in *Staphylococcus aureus*

Stephanie S.R. Souza, Marven J. Berlus, Cheryl P. Andam
Department of Biological Sciences, University at Albany, State University of New York, Albany, New York, USA

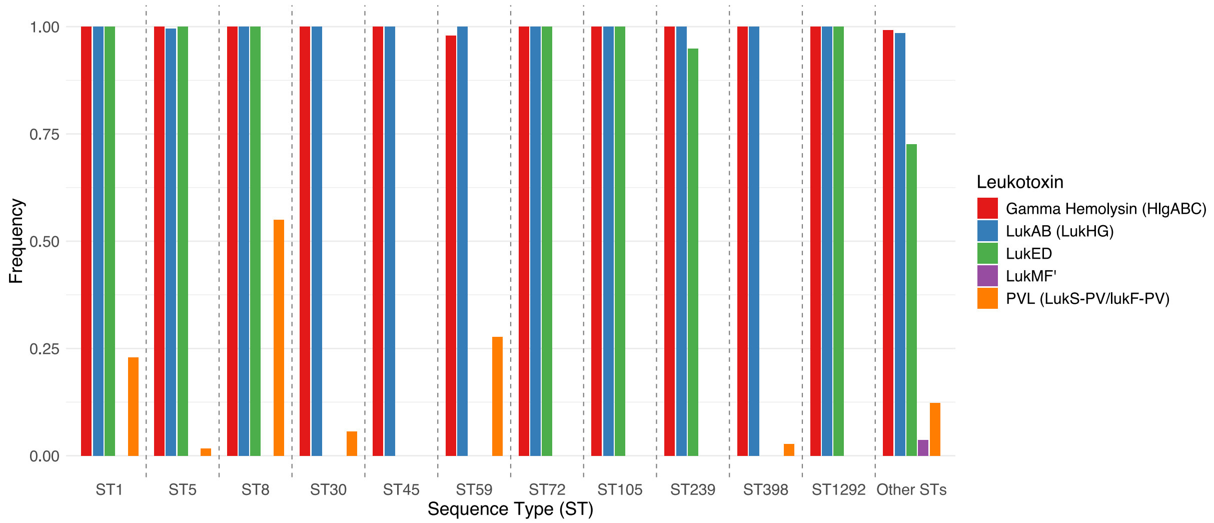

Supplementary Figure 1. Distribution of the five leukotoxin operons among the 11 most common *Staphylococcus aureus* sequence types (ST). Bar plot shows the relative frequency of each operon, represented by a different color. Less common STs are grouped together in the category "Other STs".

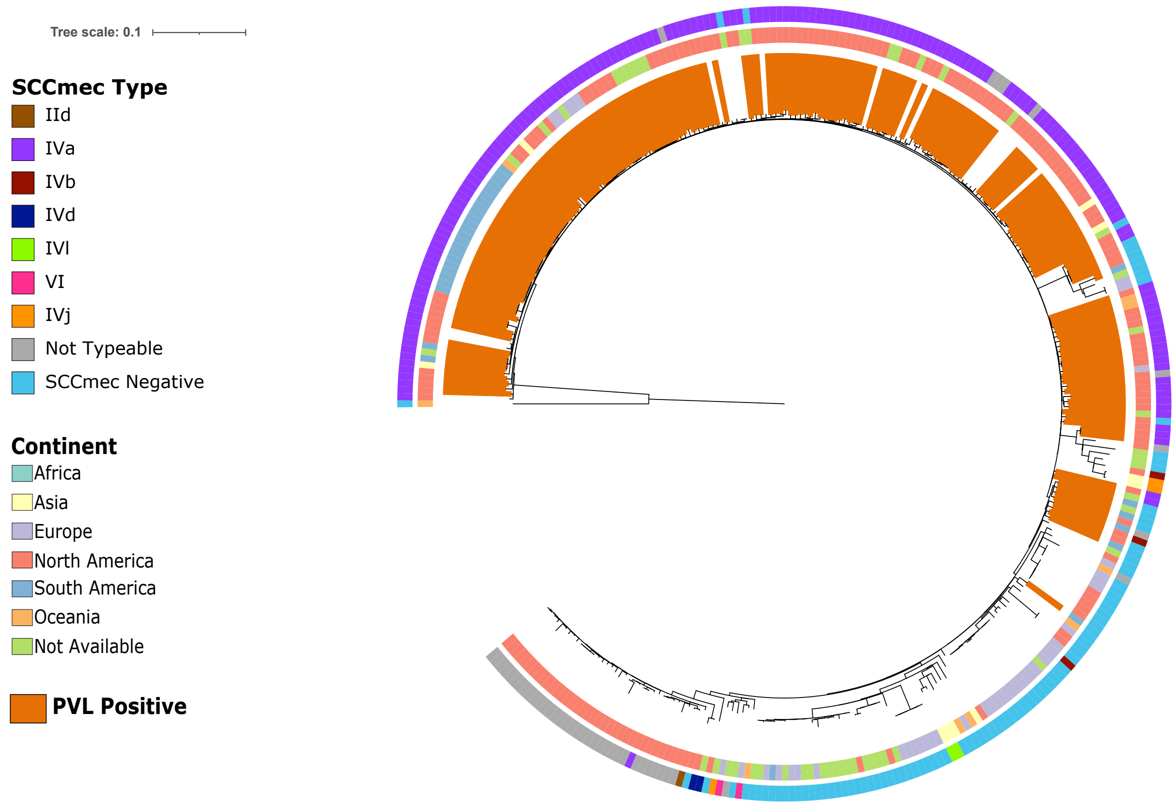

Supplementary Figure 2. Phylogenetic tree of ST8 *Staphylococcus aureus* genomes. Maximum likelihood tree showing the phylogenetic relationships of the 320 ST8 genomes. Tree scale represents the number of nucleotide substitutions per site. The tree is rooted on its midpoint. Colored lines extending out of the branches of the tree represent the presence of PVL (Panton-Valentine Leukocidin) operon. The outer rings represent continent and SCC*mec* (Staphylococcal Cassette Chromosome *mec)* type.
